# MTA1表达与中国肺癌患者预后关系的*meta*分析

**DOI:** 10.3779/j.issn.1009-3419.2017.10.04

**Published:** 2017-10-20

**Authors:** 海 钟, 云 唐, 英 王, 伟 谷

**Affiliations:** 210006 南京，南京医科大学附属南京医院呼吸科 Department of Respiration, Nanjing Hospital Afliated to Nanjing Medical University, Nanjing 210006, China

**Keywords:** 肺癌, MTA1, 预后, *Meta*分析, Lung neoplasms, MTA1, Prognosis, *Meta*-analysis

## Abstract

**背景与目的:**

已有的研究表明：在多种恶性肿瘤中发现转移相关蛋白1（metastasis associated protein 1, MTA1）发挥着促进肿瘤侵袭与转移的作用，且与肿瘤患者预后不佳有关。目前MTA1在肺癌中的预后作用仍有争议，故我们采用*meta*分析的方法评估其在肺癌患者中的预后价值。

**方法:**

通过计算机检索PubMed、Embase、万方数据库、中国生物医学文献数据库、中国知网等数据库，收集纳入研究MTA1表达与肺癌预后关系的文献及相关数据，采用Stata 12.0软件进行数据分析，合并值为风险比（hazard ratio, HR）。

**结果:**

总共纳入8项研究共712例中国肺癌患者，对这些研究进行异质性检验，发现存在异质性（*I*^2^=59.0%, *P*=0.017），故采用随机效应模型进行数据合并得HR=2.07（95%CI: 1.42-3.02, *P* < 0.001）。同时分层分析显示在非小细胞肺癌（non-small cell lung cancer, NSCLC）中各研究无明显异质性（*I*^2^=47.0%, *P*=0.093），采用固定效应模型合并得HR=1.66（95%CI: 1.27-2.18, *P* < 0.001）。

**结论:**

MTA1高表达可能是中国NSCLC患者预后不良的一个指标，在肺癌及小细胞肺癌中的预后价值尚缺乏证据。

## Abstract (en)

**Background and objective:**

It has been proven that metastasis associated protein 1 (MTA1) was correlated with tumor invasion and migration and poor prognosis of several malignant carcinomas. The prognostic role of MTA1 expression in lung cancer remained controversial, we performed this *meta*-analysis to assess the prognostic value of MTA1 expression in lung cancer.

**Methods:**

A systematic literature search was conducted to identify eligible studies which dealing with the relationship between MTA1 expression and prognosis of lung cancer in PubMed, Embase, Wanfang databases, China Biology Medicine disc and China National Knowledge Infrastructure. The pooled hazard ratio (HR) were used to assess the relationship between MTA1 expression and prognosis of lung cancer. All data were analysed using the Stata 12.0 software.

**Results:**

A total of 712 Chinese lung cancer patients from 8 studies were included in this *meta*-analysis. There was significant heterogeneity existed in these studies (*I*^2^=59.0%, *P*=0.017), so we used a random effects model to calculate the pooled HR=2.07 (95%CI: 1.42-3.02, *P* < 0.001). At the same time, we observed through stratified analysis by histology that there was no significant heterogeneity in non-small cell lung cancer (NSCLC), and the fixed effects model was used to calculate the pooled HR=1.66 (95%CI: 1.27-2.18, *P* < 0.001).

**Conclusion:**

Overexpression of MTA1 could be a marker of poor prognosis in Chinese NSCLC patients, but not in lung cancer or small cell lung cancer. Additional studies with larger samples and well-designed were required to derive the more precise outcome.

目前，肺癌仍然是严重威胁人类健康和生命的恶性肿瘤之一。根据我国最新的癌症统计数据^[1]^显示，肺癌是最常见的新发癌症和最主要的癌症死亡原因之一。尽管肺癌的诊断及分子靶向治疗有了较大发展，但是肺癌的预后仍然较差，5年生存率没有显著提高^[2]^。因此，对肺癌患者预后因素的研究有助于对患者的预后进行有效的判断，同时有利于个体化治疗方案的制定。转移相关蛋白1（metastasis associated protein 1, MTA1）是肿瘤转移相关蛋白家族成员之一，其编码基因位于染色体14q32.3，是一种分子量为80 kDa的核蛋白^[3, 4]^。多项研究发现，MTA1在乳腺癌^[5]^、结肠癌^[6]^、食管癌^[7]^、胃癌^[8]^、肝癌^[9]^、肺癌^[10]^等肿瘤组织中呈高表达，对肿瘤的发展、转移起着重要的作用。同时，多项临床研究分析了MTA1与肺癌的预后关系，但是对其预后价值判断仍存在较大争议。有研究报道MTA1高表达是肺癌患者预后的危险因素^[11]^，有研究则认为MTA1不影响肺癌患者的预后^[12]^。为了减少各项研究间的偏倚与差异，我们采用*meta*分析的方法综合评估MTA1表达在肺癌患者中的预后价值。

## 1 材料和方法

### 1.1 检索方法及策略

通过计算机检索PubMed、Embase、万方数据库、中国生物医学文献数据库、中国知网等数据库收集相关文献，截止时间为2017年6月。以“Metastasis associated protein 1或MAT1”和“lung carcinoma或lung cancer或lung tumor或lung neoplasm”为检索词在PubMed、Embase数据库检索，检索语言为英文；以“转移相关蛋白1或MTA1”和“肺癌”为检索词在万方数据库、中国生物医学文献数据库、中国知网等数据库中进行检索，检索语言为中文。

### 1.2 纳入和排除标准

纳入标准：①研究对象为通过病理或细胞学确诊的肺癌患者；②研究中包含了MTA1与肺癌总生存期（overall survival, OS）的关系；③研究直接或间接提供了HR及其95%CI的数据；④当来自同一患者群的研究数据在不同的期刊上发表时，我们选择最完整或最新的研究；⑤纳入文献仅限于已公开发表的全文，所有数据从原文中获取。排除标准：①研究未能提供有关生存率方面的资料或数据；②为非原发性肺癌如复发癌或者转移性肿瘤。

### 1.3 数据提取

两名研究者独立进行文献的筛选，后进行交叉审核，当出现意见不一致时通过讨论最后达成一致。同时提取每篇文献中的下列信息：第一作者、发表时间、国家、研究纳入的病例数、肿瘤的病理类型、临床分期、随访时间、检测方法、HR及其95%CI。

### 1.4 质量评估

两名研究者使用Newcastle-Ottawa scale（NOS）评分方法对所纳入的文献进行质量评估。在NOS评分中，每项研究主要通过3个方面进行评估：队列的选择、队列的可比性、结果的评估。在队列的选择方面最多可得4分，队列的可比性最多可得2分，结果的评估最多可得3分。研究得分越高说明质量越好。

### 1.5 统计学方法

采用*meta*分析的方法对所纳入研究的HR及其95%CI进行合并处理，并绘制森林图。同时采用*Q*检验与*I*^2^统计量进行异质性检验，当*P* < 0.05或*I*^2^ > 50%时提示存在明显的异质性，采用随机效应模型进行结果合并；当*P* > 0.05或*I*^2^ < 50%显示无明显异质性，采用固定效应模型进行结果合并^[13]^。合并的HR > 1表明高表达MTA1是肺癌预后不利的因素，而HR < 1则相反。使用漏斗图、*Begg's*法和*Egger's*法进行发表偏倚的检测，*P* < 0.05提示存在明显的发表偏倚^[14, 15]^。通过逐一剔除单个研究进行敏感性分析以确认结果的稳定性。所有数据的统计分析均使用Stata 12.0软件完成。

## 2 结果

### 2.1 文献检索及特征

通过上述检索策略共检索到295篇文献。经过阅读文献标题、摘要，排除274篇文献，剩下的21篇文献通过阅读全文进行排查。阅读全文后，总共有8篇文献符合研究的纳入与排除标准^[10-12, 16-20]^。文献检索流程见图 1。所纳入的8篇研究中共有712例中国肺癌患者，实验标本均为肺癌组织切片，均采用免疫组化方法进行MTA1表达的检测。同时在数据提取中，若文献同时提供了单变量分析和多变量分析的结果，取多变量分析结果，因为多变量分析排除了有关混杂因素，更为准确。其中4篇研究提供了完整的HR及其95%CI数据，3篇研究可根据文献提供的数据利用Parmar等^[21]^的方法计算HR及其95%CI^[12, 17, 20]^，1篇文献的HR数据根据生存曲线图获得^[19]^。各研究的基本特征见表 1。

**1:**
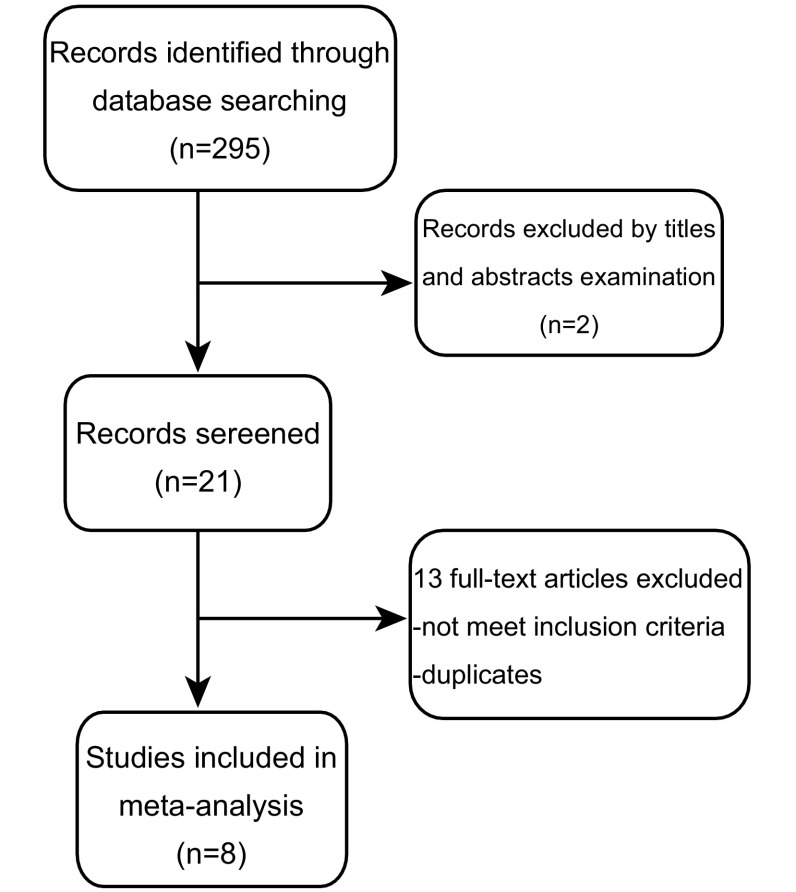
文献检索流程图 Flow chart of search strategy and study selection

**1:** 所纳入*meta*分析文献的基本特征 Main characteristics of the included studies in our *meta*-analysis

First author	Year	Country	Patient number	Histology	Stage	Follow-up time (mo)	Analysis of variable	HR (95%CI)	NOS
Ma K	2017	China	54	NSCLC	Ⅰ-Ⅳ	96-120	Multivariate	1.820 (0.717-4.618)	7
Li S	2016	China	125	NSCLC	Ⅰ-Ⅲ	16-90	Multivariate	1.300 (0.773-2.256)	7
Zhou N	2015	China	118	NSCLC+SCLC	Ⅰ-Ⅲ	> 60	Multivariate	4.960 (2.400-10.250)	7
Li SH	2011	China	102	NSCLC	Ⅰ	16-110	Multivariate	2.170 (1.105-4.247)	8
Yu Y	2010	China	60	NSCLC	Ⅰ	12-64	Multivariate	5.226 (1.575-17.338)	7
Zhang N	2007	China	101	NSCLC	Ⅰ-Ⅳ	1-101	Multivariate	1.040 (0.610-1.780)	6
Chen YT	2014	China	52	SCLC	-	26-62	Univariate	1.910 (1.030-3.540)	6
Zhu X	2010	China	100	NSCLC	Ⅰ-Ⅳ	1.4-59.3	Univariate	2.452 (1.324-4.543)	6
NSCLC: non-small cell lung cancer; SCLC: small cell lung cancer; NOS: Newcastle-Ottawa scale.									

### 2.2 *Meta*分析结果

所纳入的8篇研究中，3篇报道MTA1表达与肺癌患者的预后无关，5篇报道MTA1高表达是肺癌预后的危险因素。对纳入研究进行汇总分析，异质性检验提示存在明显的异质性（*I*^2^=59.0%, *P*=0.017），故采用随机效应模型进行结果合并的HR=2.07（95%CI: 1.42-3.02, *P* < 0.001），见图 2A。异质性的原因可能与分析变量不同、临床分期、病理类型、纳入病例数、文献评分等因素有关，故进一步进行分层分析，具体结果见表 2。其中针对肿瘤病理类型进行亚组分析显示，在非小细胞肺癌（non-small cell lung cancer, NSCLC）中各研究无明显异质性（*I*^2^=47.0%, *P*=0.093），合并的HR=1.66（95%CI: 1.27-2.18, *P* < 0.001），见图 2B；针对病例数进行亚组分析显示，在病例数≤100的各研究中无异质性（*I*^2^=0, *P*=0.488），合并的HR=2.29（95%CI: 1.58-3.34, *P*=0.001）。

**2:**
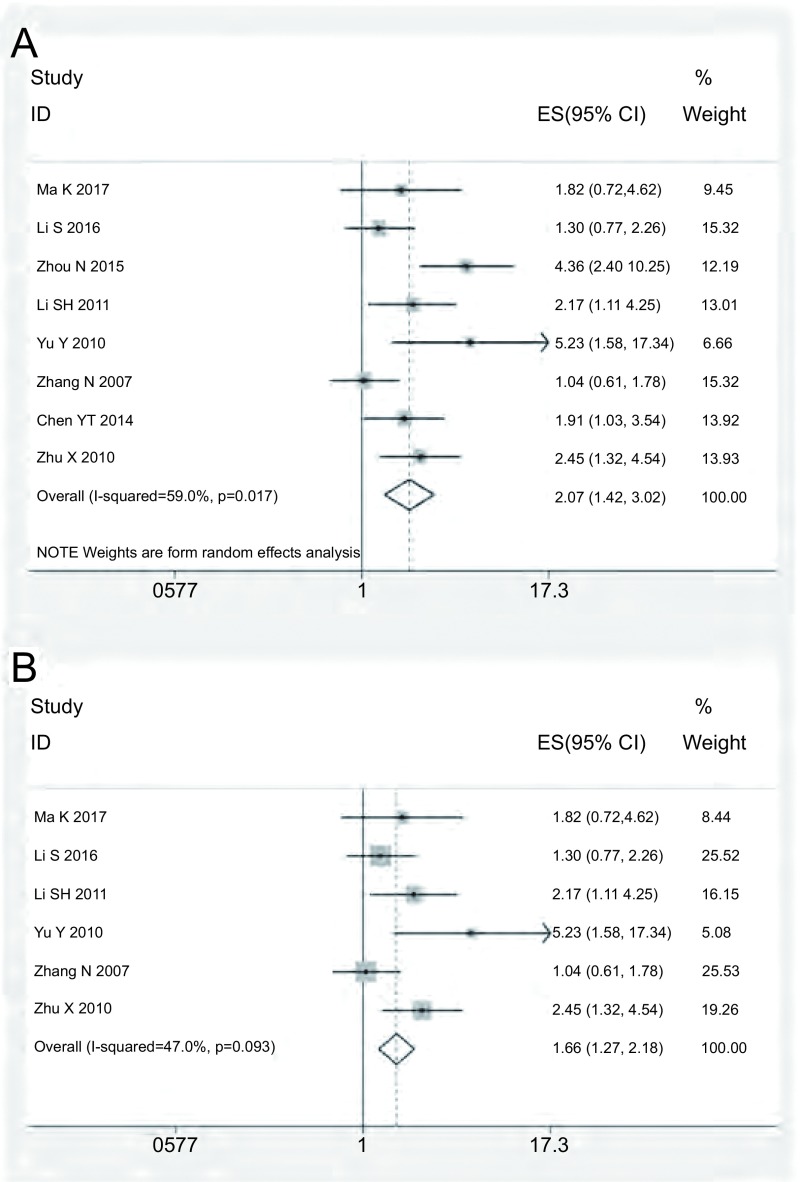
在肺癌（A）与NSCLC（B）中总生存期合并后的HR的森林图 Forest plot showing the pooled HR for overall survival in lung cancer (A) and NSCLC (B)

**2:** 以不同变量对肺癌中MTA1表达与总生存期关系进行分层分析 Stratifiedanalysis of MTA1 expression and overall survival in lung cancer

Variable	No. of studies	HR (95%CI)	*P*	Heterogeneity		Model used
				*I*^2^	*P*_h_	
Analysis of variable						
Multivariate	6	2.09 (1.23-3.54)	0.006	69.30%	0.006	Random
Univariate	2	2.16 (1.40-3.35)	0.001	0	0.575	Fixed
Tumor stage						
Ⅰ	2	2.68 (1.49-4.82)	0.001	36.30%	0.21	Fixed
Ⅰ-Ⅳ	5	1.92 (1.12-3.28)	0.018	71.20%	0.008	Random
Histology						
NSCLC	6	1.66 (1.27-2.18)	< 0.001	47.00%	0.093	Fixed
NSCLC+SCLC	2	3.02 (1.19-7.68)	0.021	74.00%	0.05	Random
Patient numbers						
> 100	4	1.88 (1.00-3.56)	0.051	77.00%	0.005	Random
≤100	4	2.29 (1.58-3.34)	0.001	0	0.488	Fixed
Quality score						
> 6	5	2.47 (1.41-4.34)	0.002	62.60%	0.03	Random
≤6	3	1.66 (0.99-2.79)	0.054	56.90%	0.098	Random

### 2.3 发表偏倚与敏感性分析

我们使用漏斗图、*Begg's*法和*Egger's*法进行发表偏倚检测，根据漏斗图显示在肺癌与NSCLC中均未见明显的不对称，见图 3。其中，在肺癌中由*Begg's*法所得*P*=0.108及*Egger's*法所得*P*=0.077，在NSCLC中由*Begg's*法所得*P*=0.133及*Egger's*法所得*P*=0.091，均提示无明显的发表偏倚。通过逐一剔除单个研究进行敏感性分析以检验结论的稳定性，结果显示在肺癌和NSCLC中结论均无明显的改变，提示结论较为稳定、可信，见图 4。

**3:**
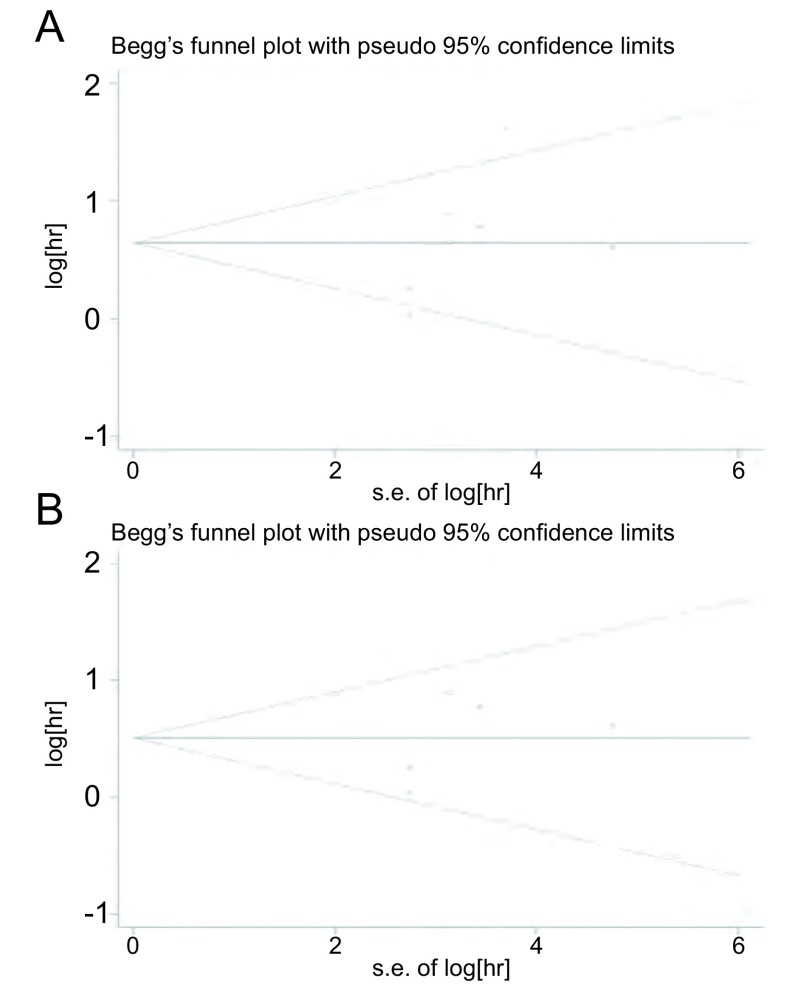
*Begg's*漏斗图检测在肺癌（A）与NSCLC（B）中潜在的发表偏倚 *Begg's* funnel plot to assess potential publication in lung cancer (A) and NSCLC (B)

**4:**
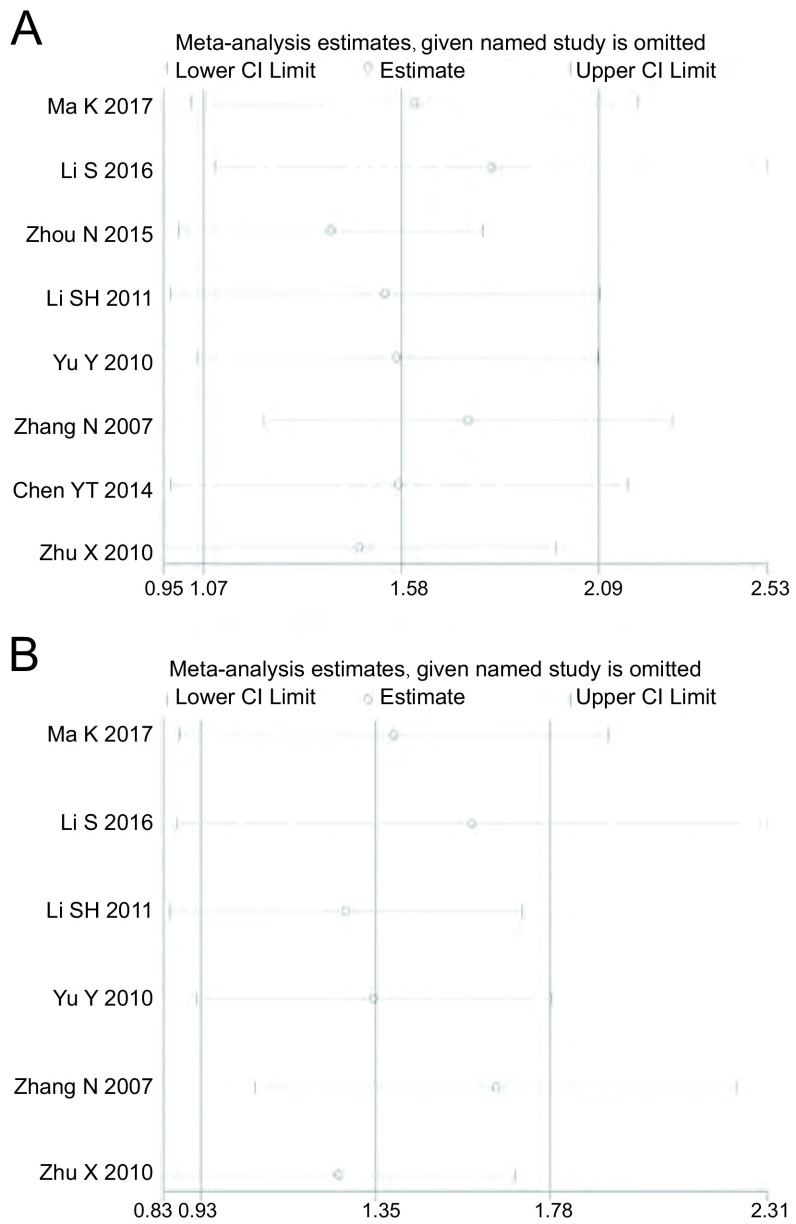
敏感性分析评估在肺癌（A）与NSCLC（B）中所得结论的稳定性 Sensitivity analysis of included studies on assessment the reliability of our conclusionin lung cancer (A) and NSCLC (B)

## 3 讨论

肿瘤转移是恶性肿瘤的重要生物学特征，也是目前肺癌临床治疗的难题。临床研究表明，肿瘤侵袭与转移是90%肺癌患者死亡的主要原因^[22]^。多项研究发现MTA1是肿瘤转移的重要调节因子，不仅是某些基因启动子的共激活因子，包括β-链蛋白（β-catenin）、乳腺癌扩增序列3（BCAS3）和信号转导及转录激活因子3（STAT3），也可作为一些靶基因的转录共阻遏因子，如E-钙粘蛋白^[23]^。同时，MTA1在多种恶性肿瘤如乳腺癌、结肠癌、食管癌、胃癌、肝癌中提示有一定的预后价值，但是在肺癌中的预后作用仍有争议^[24]^。

本研究采用*meta*分析的方法评估MTA1高表达在肺癌患者中的预后价值，共纳入8篇文献，且研究对象均为中国肺癌患者，未检索到符合条件的含其他国家肺癌患者的研究。对这些研究进行异质性检验，发现存在异质性（*I*^2^=59.0%, *P*=0.017），故我们采用随机效应模型进行结果合并的HR=2.07（95%CI: 1.42-3.02, *P* < 0.001），这表明MTA1高表达可能是中国肺癌患者不良预后的指标。由于存在明显的异质性，故我们采用分层分析进一步确定异质性的来源。根据各研究全文数据的收集，我们依据分析变量不同、肿瘤病理分期、病理类型、纳入病例数、文献评分等因素进行分层分析。其中针对肿瘤病理类型进行亚组分析显示，在NSCLC中各研究无明显异质性（*I*^2^=47.0%, *P*=0.093），合并的HR=1.66（95%CI: 1.27-2.18, *P* < 0.001），提示MTA1高表达也是NSCLC预后不良的标志物。同时在病例数≤100的各研究中无异质性（*I*^2^=0, *P*=0.488），合并的HR=2.29（95%CI: 1.58-3.34, *P*=0.001）。而依据分析变量不同、肿瘤病理分期、文献评分等因素进行分层分析，仍发现较明显的异质性，故所纳入研究的肺癌患者病理类型及病例数不同可能是异质性的主要来源。且通过发表偏倚检测未见明显的发表偏倚，敏感性分析检测结论在肺癌与NSCLC中均较为稳定。

MTA1高表达可作为肺癌患者的不良预后的标志可从其所具有的功能得到诠释。MTA1是核小体重塑及组蛋白去乙酰化酶复合物（nucleosomeremodeling and histone deacetylase, NuRD）的重要组成部分^[23]^。MTA1与组蛋白去乙酰化酶结合后可转移至目标基因的启动子区域，去除组蛋白的乙酰基从而改变染色体的状态，影响目的基因的转录过程，如抑制抑癌基因乳腺癌1号基因、p21^WAF1^、肿瘤超甲基化基因1等的转录，从而促进肿瘤的发生^[23]^。同时，含有MTA1的组蛋白去乙酰化酶1复合物可以使非组蛋白如p53及缺氧诱导因子1α去乙酰化，其中p53去乙酰化后可减弱由其诱导的细胞凋亡作用，而缺氧诱导因子1α去乙酰化后可增强自身的稳定性，进而促进肿瘤血管生成，这些均可促进肿瘤发展^[25, 26]^。而且，研究发现MTA1可促进肿瘤细胞上皮间质转化（epithelial-mesenchymal transition, EMT）进而促进肿瘤的转移：MTA1作为转化生长因子β1的下游效应蛋白可抑制上皮钙粘蛋白即E-钙粘蛋白的表达，从而促进EMT^[27]^；在卵巢癌细胞中还发现MTA1可激活转录因子Snail与Slug，从而抑制E-钙粘蛋白的转录，进而促使EMT的发生^[28]^；同时，在NSCLC中发现MTA1可通过激活AKT/GSK3β/β-catenin信号通路增加N-钙粘蛋白、减少E-钙粘蛋白促进EMT^[10]^。由于MTA1在肿瘤的发生及发展、转移、血管生成中发挥着重要作用，故MTA1高表达可能成为肺癌预后的不良标志。

本研究还存在一些局限性：①只有8篇临床研究共712例中国肺癌患者纳入我们的*meta*分析中，且研究对象主要以NSCLC为主，仅有1篇为小细胞肺癌（small cell lung cancer, SCLC），另一篇为NSCLC+SCLC，故MTA1在中国肺癌及SCLC患者中的预后价值仍需要更多、更详实的研究来进一步证实。②所纳入的研究均采用免疫组化方法来检测MTA1表达情况，但所使用的抗体厂家、抗体稀释浓度、分析方法及阳性结果判断标准均不同，这些均可能导致实验结果的不同。③由于我们所收集的研究数据仅从原文中获得，无法获取原始数据，故无法以淋巴结转移情况、PS评分、治疗方案等影响肺癌预后较确定的因素作为分层因素，来进一步分析MTA1高表达与肺癌的预后关系。④本研究的检索语种限定为中文和英文，而其他语种如德语、法语等研究未包括在内，这可能引起不可避免的偏倚。⑤虽然我们的研究未发现明显的发表偏倚，却不能完全避免。这是因为阳性研究结果更容易被杂志接受发表，而阴性研究结果更易被拒绝甚至不发表。

综上所述，本研究通过*meta*分析的方法对相关研究进行综合性分析发现MTA1高表达可能是中国NSCLC患者预后不良的一个指标，但是在肺癌及SCLC中的预后价值尚缺乏证据。同时，由于本研究存在上述局限性，研究结论还需要大样本、多中心、前瞻性随机对照研究进行证实。
